# Supplementary material for: Automated detection of superficial fungal infections from microscopic images through a regional convolutional neural network
Source: PLoS One. 2021 Aug 17;16(8):e0256290. doi: 10.1371/journal.pone.0256290 (PMC8370604; doi:10.1371/journal.pone.0256290)
Supplement: Attachment — Submitted filename: Response_to_Reviewer.docx [file pone.0256290.s001.docx]

Response to Reviewer

Dear Reviewer

We greatly appreciate your thoughtful comments that helped us improve the manuscript.

We trust that all your comments have been addressed accordingly in a revised manuscript. Thank you very much for your effort.

**1. Please ensure that your manuscript meets PLOS ONE's style requirements, including those for file naming.**

-> We modified the manuscript to suit PLOS ONE’s style requirements. Thank you!

**Reveiwer #1**

-**In my view the paper provides a valid and valuable contribution towards a more efficient detection of fungal infections, which is certainly not a trivial task. The achieved results are good, nevertheless I see that one of the major limitations that the authors did not dwell into, is that the current results are based on images collected from a single imaging and sample preparation setup and in this way, it's difficult to see whether the proposed model would achieve similarly great results on a more general basis.**

->

The sample preparation setup and images used in our research are not just simple images created artificially for research. They are real images from microscope settings used in real-world practice at dermatology clinics. Dermatologists read images obtained from the same settings as our paper, so we believe the results meet that level of generalizability.

-**Actually, there is a tale-tell sign in the paper regarding this problem, that is, when mixing the datasets with 40x and 100x magnification, there is a significant drop in precision and F1 scores. For this reason I would like to see that that the authors discuss this issue further, particularly in the sense that what could be expected (or what modifications are anticipated in their method) in case they applied the deep-learning based autodetection on a more complete set of images that originate from various imaging setups and settings, as well as taking into account potential variability in sample preparation.**

**For this very reason I feel that the authors need to be more cautious with a conclusion that the presented results demonstrate a higher sensitivity and specificity than those achieved by manual detection of experts, since the experts can clearly gather practice on a wide variety of setups with differing sample/image quality.**

->

As you mentioned, the mixed datasets (100 + 40)x showed a lower detection performance than the individual data sets. To increase the performance, we believe a larger mixed dataset is required. However, it was not easy to increase the dataset during the research.

The goal of our research is to develop an automatic hyphae detection machine to accelerate hyphae testing speed in the field. We will develop a device that can automatically inspect slide images with a variable magnification from 100x to 40x. We scanned the whole slide at 40x magnification, and then we zoomed in to 100x for the area suspected of hyphae. Based on this background, we tried to compare the search rates for the 40x and 100x magnifications, and the mixed data set was analyzed with the network. YOLO is one of the efficient networks for object detection. However, it has a drawback: the accuracy of small object detection is lower than that of big-sized object detection. By analyzing the three different datasets, we concluded that we should make multiple networks for 40x and 100x magnification to maximize the performance.

As you have pointed out, we also agree with your opinion about the proposed method having lower performance in detecting hyphae than a well-trained expert. However, it has been verified through many studies that the recognition rate of the YOLO network is as sufficiently high as that of humans. Through the results of this study, it was confirmed that the precision and reproducibility were similar to those of a well-trained expert even in the hyphae detection problem. In future, if additional learning is performed through continuous data accumulation, we believe that the performance will continue to improve.

(1) **The authors use IOU=0.25 value as reference, while it is well-known that an IOU of at least 0.5 is considered a good result in object detection. Therefore, I contend that using IOU=0.25 needs some more specific justification in the paper.**

->

We believe that the determination of the threshold of the IOU should be applied differently depending on the problem. In general, the three values 0.25, 0.5, and 0.75 are used as cutoff values in object detection studies. Our study evaluated the performance of all the three values since we first applied the object detection algorithm for hyphae. Our model detects hyphae only in the form of a box, but hyphae have a characteristic that it cannot be displayed in accordance with the shape of the box because of its curved linear structure. To reduce the false-negative rate, the model should be able to detect as many hyphae suspect areas as possible. Therefore, in this study, to increase the search rate, the IOU value is set to the lowest 0.25. We have added this content to the manuscript as follows:

(Lines 166–177)

In general, the three IOU values of 0.25, 0.5, or 0.75 are used as cutoff values in object detection studies. Our study evaluated the performance of all three values since we first applied the object detection algorithm for hyphae. Our model detects hyphae only in the form of a box, but the hyphae have a characteristic form that cannot be displayed in accordance with the shape of the box because of its curved linear structure. Importantly, to reduce the false-negative rate, the model should be able to detect as many hyphae suspect areas as possible. Therefore, in this study, to increase the search rate, the IOU value was set to the lowest, 0.25. When the IOU was set to 0.25, the recall value of the 100× data model was the highest at 0.93, and the F1-score and AP values were 0.84 and 92.08, respectively. Further, the 40× data model exhibited the excellent performance with the recall, F1-score, and AP values of 0.83, 0.83, and 88.07, respectively. The (100+40)× magnification model exhibited a significantly lower performance than the other two magnification models. The performance of classification, which determines the microscopic image as positive or negative, was evaluated using the ROC curve and area under curve (AUC) values under the setting of the IOU value at 0.25.

(2) **Pg. 6, lines 140-141, it is written that: "After calculating the minimum, maximum, and average probabilities, we obtain the final probability for a given microscopic image." Here and throughout the subsequent evaluation of results it is not clear what 'final probability' means and how the authors arrive at it from the defined min, max and avg probabilities. By the same token, the usage of these probabilities seems rather vague throughout the paper and it does not stand out clearly what is the purpose of these min, max and avg probabilities. Could you please clarify?**

->

The sentence “After calculating the minimum, maximum, and average probabilities, we obtain the final probability for a given microscopic image.” leads to misunderstand how the final probability can be obtained using Pmax, Pmin, and Pavg, which are types of final probabilities, respectively. We attempted to evaluate which value is the best final probability through ROC analysis using Pmax, Pmin, and Pavg as their respective final probabilities. We have deleted the misleading sentences and corrected the contents as follows. In addition, we have added content to help understand the process of determining the optimal setting values (IOU, Pmax, threshold value) of our YOLOv4 model in the “Result” section of the manuscript.

(Lines 146–147)

(Before)

After calculating the minimum, maximum, and average probabilities, we obtain the final probability for a given microscopic image

(After)

In this study, Pmin, Pmax, and Pavg were applied as the final probability separately and evaluated as to which value showed the highest performance.

(Lines 179–181)

Between the three types of final probabilities (P_max_, P_min_, and P_avg)_, the highest performance was achieved when the final probability was set to P_max_.

(Lines 183–184)

The threshold value was calculated as 0.244 by analyzing the ROC curve. We use perfcurve function in MATLAB for ROC curve analyzing

(3) **Pg. 7. lines 166-167: "The optimal setting of the Fungus hyphae database YOLO v4 model occurs when the IoU value is 0.25 and the detection probability threshold value is 0.244". How do you arrive at the optimal probability threshold 0.244?**

->

The probability threshold proposed in our study is the result obtained by analyzing the ROC value using the MATLAB perfcurve function based on the detection result. The perfcurve function calculates the optimal detection probability threshold by analyzing the ROC curve graph to maximize performance. There are three ROC analysis based on Pmax, Pmin, and Pavg. We evaluated all probability thresholds for Pmax, Pmin, and Pavg. The threshold value obtained when analyzing the ROC value using the MATLAB perfcurve based on Pmax is 0.244. We have added this content to the manuscript as follows:

(Lines 149–152)

The probability threshold proposed in our study is the result obtained by analyzing the ROC value using the MATLAB perfcurve function based on the detection result. The perfcurve function calculates the optimal detection probability threshold by analyzing the ROC curve graph to maximize performance.

**A minor note: please provide a proper table legend for Table 2, where all abbreviated measures are clearly given in text (e.g. TP, FP written as true and false positives, which are nowhere else referenced).**

->

The legend in Table 2 was added as follows.

IOU: Intersection over union, TP: True positives, FP: False positives, FN: False negatives, AP: Average precision, AUC: Area under curve, ROC: Receiver operating characteristics

**Reviewer #2**

**- You mention how many images are contained in the data set, but not how many different skin samples (each of which probably leading to several images). This needs to be clarified. If training and test data contain images taken from the same sample, we would overestimate the performance.**

**For a real application, the model would need to generalize to completely new samples. Also, you write that samples from both skin and nails were used. Are both distributed equally among training and test data?**

->

Two dermatologists read the samples and assigned them to positive and negative classes. A total of 38 samples were collected from 38 patients, of which 10 positive cases (6 skins, 4 nails), 10 negative cases were at 40× magnification, 8 positive cases (6 skins, 2 nails), and 10 negative cases were at 100× magnification. The positive samples were divided into two groups, training and testing datasets (6 training samples and 4 testing samples were at 40× magnification; 5 training samples and 3 testing samples were at 100× magnification). As presented in Table 1, the training and testing data images were acquired from the sample obtained using this method. Therefore, training and test images were not obtained from the same sample. The image data used for training and those used for testing did not overlap. Hence, we believe that there was no performance overestimation. In addition, skin image data are more distributed than nail image data. However, this is thought to be the result of including several parts of the skin (trunk, arm, leg). We have added the content to the manuscript as follows:

(Lines 93–99)

Two dermatologists read the samples and assigned them to positive and negative classes. A total of 38 samples were collected from 38 patients, of which 10 positive cases (6 skins, 4 nails), 10 negative cases were at 40× magnification, 8 positive cases (6 skins, 2 nails), and 10 negative cases were at 100× magnification. The positive samples were divided into two groups, training and testing datasets (6 training samples and 4 testing samples were at 40× magnification; 5 training samples and 3 testing samples were at 100× magnification). As presented in Table 1, the training and testing data images were acquired from the sample obtained using this method.

**- Yolo is a standard object detection network, but by far not the only network suitable for this task. It would clearly strengthen the evaluation if you could report the performance of another method, e.g. a two-stage detector such as Mask R-CNN, as a reference.**

->

As you pointed out, we also expect that the two-stage detector could perform better than the YOLO network used in this study. However, the performance of YOLO v4 is enough to detect hyphae in real field samples. A more important consideration in selecting a network is portability and speed of detection. It is well known that YOLO is one of the fastest object detection networks and is easy to transfer embedded systems without a lot of model change. The typical two-stage detector is slower than the YOLO network and needs a lot of effort to transfer embedded systems. As we mentioned in the previous answer, the goal of our research is the development of an automatic hyphae detection system that can be utilized in the field besides a well-trained person. While examining the performance of the YOLO network for the hyphae dataset, we had in mind trying another two-stage network if the performance of YOLO had proved poor. However, we were convinced that the performance was enough therefore we did not consider an additional detection network.

**- "We also created dataset-N (100, 40, all), which included microscopic images without dermatophyte hyphae, for testing." Does this mean that the negative cases that are available for 40 and 100 are only used during training and not part of the test data?**

->

The negative case images were not used for training and were only used for testing. Due to the nature of the YOLO network, it is difficult to learn images that do not contain objects, so training about negative images was not done.

**- "All dataset": Pooling images from different magnifications is not such a practically relevant scenario. In an automated system, all images would be recorded at the same mangnification (or, if different magnifications are supported, one would have separate training sets). I would rather analyze the effect of including/excluding negative cases that will likely occur in a real application.**

->

We considered two magnifications (i.e., 40x, 100x) in this study because we created a variable magnification microscope system that adjusts automatically. If one scans a slide at a magnification of 100x, detecting the entire slide takes a long time. Therefore, we plan to create a system that first scans at 40× magnification and then scans the slide at 100× magnification where suspected hyphae would have been observed. For this reason, we conducted a study to determine whether one network can detect well even when images of two magnifications are mixed. In addition, clinicians read the slides mixed with positive and negative cases in the real-world practice. Therefore, we tested the images including negative cases.

**- The images in Fig.3 differ a lot in their appearance/color. Can you discuss this and maybe provide a few more example images in a supplementary figure? Can you show that positive/negative cases do not differ with respect to their background color, but only with respect to the existence of the target objects?**

->	There are two reasons for differences in image color. The first is the background color that occurs when recording microscopic images, and the second is the color difference that occurs in the process of converting video images into still images. Since the YOLO network standardizes all the colors in the perceived image, we believe the color differences had no effect.

**- Do all hyphae for x100 magnification look like in Fig. 3a and do all hyphae for x40 look like in Fig. 3b? Or is there more variability within x100 and x40, respectively. Again, showing more example images would help.**

->

There is a difference in size and no difference in shape that is based on or caused by the magnification. We added more images for further clarity. (We also added more example of positive images for each magnification in “Supporting information.”)

(1) x100 magnification (positive)

(2) x100 magnification (negative)

(3) x40 magnification (positive)

(4) x40 magnification (negative)

**- Also, does Fig. 3 show full resolution images or can you maybe show them at a higher resolution?**

**The hyphae in Fig. 3b are really hard to see, and some are actually covered by the labels/boxes: Can this be changed?**

->

The labeling box is a drawn line shaped as a box to show the detection results. Currently, the label is difficult to erase, so we will replace it with an image that does not interfere with identification.

**- Can you clarify how the data was recorded? What does it mean that "the images were recorded by rotating the screen at a 360 degrees angle where the hyphae were observed"? Why do you record a video instead of still images?**

->

We had to use as many images as possible to train our model to find hyphae using deep learning. However, a limitation exists because the slide sample had to be obtained from a real-world practice. Hyphae are a structure that shows a simple pattern with a long tubular structure, although the shape vary. Further, the model recognizes each image as a different image even if the same image is rotated. Therefore, one image was first recorded while rotating 360 degrees, and we converted the recorded videos into individual images. Recording videos and converting them into individual images is more efficient to obtain data than shooting each still image.

**- You claim, already in the abstract, that the network performed better than human experts. In the discussion it then becomes clear that this statement is based on a comparison to values reported in a different context by Jacob et al. Is this really comparable? It is ok to mention their results as a reference, but I would be careful making strong claims in the abstract that are then not fully supported by the results in the paper. In order to make such a claim, you would need an independent ground truth for your data, which would then serve to judge both the network and the human experts.**

->

As pointed out, in this study, we did not directly compare the accuracy of detection capacity between the experts and the model. We compared the accuracy by citing Jacob's paper, but we agree that it is not an exact comparison because it was not compared with the same slide image. In the abstract, we corrected this by writing only that the function was good, excluding the other content as follows.

(Lines 29–30)

(Before) The performance of our model was higher than the sensitivity and specificity of the known experts, indicating that it is possible to detect hyphae with reliable accuracy.

(After) The performance of our model had high sensitivity and specificity, indicating that hyphae can be detected with reliable accuracy.

**- The annotated image data set is a valuable contribution of this work and will be of interest for the image analysis community (target objects in front of complex background/also negative examples provided, which is rare). If you plan to publish images and annotations along with the paper (as indicated in the data availability section), I would mention this in the paper, referring to the respective supplementary file/web link.**

->

As you pointed out, databases are very important in deep learning research. After reviewing legal issues, we will share databases through GitHub. When this paper is published, we will add a database link to the final paper.

**- Minor points:**

**- reference [14] contains two papers and should be split**

-> We deleted one of the references (reference 7 number 14). Thank you for your meticulous attention.

**Fig 4: "contusion matrix": ouch, better write "confusion matrix”**

-> The 'contusion matrix' has been modified to 'confusion matrix'. Thank you for your meticulous attention.

***** Additional information**

We have added an image to help you understand the functional system equipped with our model currently under development. (as below):
