# Supplementary material for: Automated detection of superficial fungal infections from microscopic images through a regional convolutional neural network
Source: PLoS One. 2021 Aug 17;16(8):e0256290. doi: 10.1371/journal.pone.0256290 (PMC8370604; doi:10.1371/journal.pone.0256290)
Supplement: Attachment — Submitted filename: Response_to_reviewers_FINAL.docx [file pone.0256290.s002.docx]

Response to Reviewer

Dear Edtior,

We thank the reviewers for the insightful comments and suggestions that have helped improve the manuscript significantly.

We have made several changes to the manuscript according to the suggestions of the reviewers. We appreciate the efforts put in towards the review of this manuscript.

The line numbers mentioned below are based on the “Revised Manuscript with Track Changes” file.

As mentioned earlier (at first revision), we will share the public database link after a legal review once our paper is accepted. As of now, we have made available a part of the image dataset with a label from Figshare, for review purposes—the private Figshare link is <https://figshare.com/s/b8f2f80f40b6789daff3>. Please note that this private link is only for review purposes, and will be valid for three months.

Legal reasons for data disclosure can be obtained on the basis of publication, if the manuscript is accepted. We will include the link for the complete dataset once our paper is published.

**Reviewer #1:**

**The only question I still find somewhat ambiguous, and with some claims unwarranted in my view, is the performance of the author's model in terms of (1) comparability to that of dermatologists in real-world practice and (2) whether it can be generalized for other imaging setups than the specific one used in this study. The authors themselves exhibit in their reply to the reviewers' comments that while one could expect that their model may retain good performance in such relations, there is no hard evidence for it at this stage.**

**Therefore, a cautious approach for deriving conclusions in that regard should be reflected throughout the paper.**

**Regarding (1), the authors have already softened their claim in the Abstract ("The performance of our model had high sensitivity and specificity, indicating that hyphae can be detected with reliable accuracy."). This, however, is in contrast what is written in the Discussion: line 256-257. : "The performance of our model is higher than the sensitivity and specificity of the known experts, indicating that it is possible to detect hyphae with reliable accuracy. Accordingly, diagnosis can be made more efficiently and in a more straightforward manner." I would suggest that these parts should conform to what is written in the Abstract.**

- We understand the concerns raised by the reviewer. The sentence in the ‘discussion’ part (lines 256-257 before) was modified as follows to match the content of the Abstract. Also, lines 232-233 “Clearly, both these indicators (sensitivity and specificity) exhibit higher performances than those of the known expert.” of the Discission were deleted for the same reason.

(Before) Lines 272-273

The performance of our model is higher than the sensitivity and specificity of the known experts, indicating that it is possible to detect hyphae with reliable accuracy.

(After) Lines 272-273

The performance of our model had high sensitivity and specificity, indicating that it is possible to detect hyphae with reliable accuracy.

**As for (2), I would see it necessary to insert a few sentences again in the Discussion part, where the text would make it clear that: although the applied methodology certainly demonstrates very promising performance, it has been only tested with a single imaging setup and in future work a more thorough testing is needed with a bigger dataset from more variable imaging settings in order to establish how reliable the model's performance is and how it compares to that of real-world dermatologists in such a broader context.**

- We have added the following sentences in the Discussion section to express the meaning more clearly.

Lines 253-256

Second, since our model was developed using image data that was obtained from a setting of single clinic, there is a possibility of performance decrease in different settings of various clinics. The significance of the proposed study is that hyphae can be identified through deep learning techniques. We will continue to train the autodetection model with more data from various clinics.

**Reviewer #2: The authors have addressed my comments and now provide additional information, figures and access to the data. There are still a few smaller issues that can be resolved by a minor revision:**

**- Thanks for explaining that you are developing "an automatic hyphae detection system that can be utilized in the field". I would actually mention this in the abstract and make it more clear in the introduction. This would help to introduce your application case to the reader, and it also motivates your decision to prefer a fast network over a potentially more accurate one.**

- We thank the reviewer for the encouraging words on our manuscript. The following content has been added to the Abstract and Introduction. Further, lines 64-66 of the Introduction have been deleted.

Line 31-33

We aim to develop an automatic hyphae detection system that can be utilized in real-world practice through continuous research.

Line 66-67

Based on our result, we are developing an automatic hyphae detection system that can be utilized in real-world practice through continuous research.

**-Fig. 1 illustrates the training process with a heavy multi-GPU machine. Maybe something like the image you have appended at the end of the reviewer PDF could be used to illustrate that the final system with the trained model should require only a smaller mobile device?**

- A heavy multi-GPU machine is required in the training process, but a smaller mobile device is sufficient for the final system that is equipped with the autodetection model obtained through training. A previous study showed that the YOLOv4 model used in our study can be used by attaching it to a mobile device. Figure 1 shows the overall workflow applied in this study. Although our ultimate goal is to develop a small mobile device equipped with a trained model, the results of this study was validated on a multi-GPU machine. Thus, appending the image of a small mobile device in the workflow may cause a misunderstanding among the readers that this paper includes data about the small mobile device, which is not validated in this study. Therefore, we have added the following content to the discussion part of the manuscript and have also added the corresponding reference.

Lines 266-269

Although heavy multi-GPU machine is required in the training process,, a smaller mobile device is sufficient for the final system that is equipped with the autodetection model obtained through training. A recent study showed that the YOLOv4 model used in our study can be attached to a mobile device [21].

**- I would move the new paragraph between lines 93 and 99 to the section "dataset generation"**

**You could also include the information about samples and skin/nail into Table 1.**

- We thank the reviewer for the insightful comment. The paragraph between lines 97-103 has been moved to lines 112-116 in the section “Dataset generation.” In addition, the skin/nail information has been added to Table 1 as shown below:
- Table 1. Summary of fungus hyphae dataset

| **Dataset** | **Optical Magnification Ratio (**×**)** | **Samples** | | | | | | | **Image dataset** | | | |
| --- | --- | --- | --- | --- | --- | --- | --- | --- | --- | --- | --- | --- |
|  |  | **Total** | **Positive Case** | | | | **Negative Case** | | **Total** | **Positive Case** | | **Negative Case** |
|  |  |  | **Training** | | **Testing** | | **Testing** | |  | **Training** | **Testing** | **Testing** |
|  |  |  | **Skin** | **Nail** | **Skin** | **Nail** | **Skin** | **Nail** |  |  |  |  |
| **Dataset-100** | 100 | 18 | 4 | 1 | 2 | 1 | 5 | 5 | 1279 | 660 | 440 | 179 |
| **Dataset-40** | 40 | 20 | 4 | 2 | 2 | 2 | 5 | 5 | 1621 | 595 | 398 | 628 |
| **All dataset** | 100+40 | 38 | 8 | 3 | 4 | 3 | 10 | 10 | 2900 | 1255 | 838 | 807 |

**-"We split the labeled dataset into training and test sets at a 6:4 ratio."**

**I understand this was stratified by sample and skin/nail. Apart from that, was the 6:4 split random?**

- We understand the concern raised by the reviewer. There is no significant difference between hyphae and other floats in skin and nail samples. Therefore, we split the dataset into training and testing sets at a ratio of 6:4 at random without distinction.

**- Regarding the sentence: "the images were recorded by rotating the screen at a 360 degrees angle where the hyphae were observed"**

**With you explanations, I now see what you mean. But the sentence is hard to understand and could be improved: You rotate a screen or actually a camera? Rotating by 360 degrees would mean no rotation?**

- We understand the concern raised by the reviewer. To express the meaning clearly, the sentence has been modified as follows.

(Before) Line 95-96

the images were recorded by rotating the screen at a 360 degrees angle where the hyphae were observed

(After) Line 95-96

the images were recorded over a 360° rotation of the slide where the hyphae were observed.

**- It would be helpful to also show an example with ground truth annotations. There are some dubious cases where the non-expert reader wonders whether some faint structures are true negatives or actually hyphae that were missed.**

- We thank the reviewer for the suggestion. The ground truth annotations have been added to Figure 3. This makes it easier for non-expert readers to compare whether the hyphae observed by the model is real hyphae.

(Before) Fig.3

(After) Fig.3

**Fig 3. Example images of the autodetection of hyphae with bounding box.^￥^** (A) positive case with 100× magnification, (B) positive case with 40× magnification, (C) negative case with 100× magnification, and (D) negative case (false detection) with 40× magnification.

^￥^The ground truth were marked with a green box in positive case (A,B).

**Additionally, we have made the following revision to the manuscript:**

**#1.**

We deleted Line 88 “prepared with 10% KOH” because it was duplicated with the following sentence.

**#2.**

While moving lines 93-99 to the section “Dataset generation”, the sentence “Two dermatologists read the samples and assigned them to positive and negative classes.” was moved to line 90, and the sentence “As presented in Table 1, each training and testing data images were acquired from the sample obtained in this way” lines 102-103 was deleted.
